# Subwavelength Grating Cascaded Microring Resonator Biochemical Sensors with Record-High Sensitivity

**DOI:** 10.1021/acsphotonics.4c00778

**Published:** 2024-08-01

**Authors:** Weiqing Cheng, Shengwei Ye, Bocheng Yuan, John H. Marsh, Lianping Hou

**Affiliations:** James Watt School of Engineering, University of Glasgow, Glasgow G12 8QQ, U.K.

**Keywords:** subwavelength grating, cascaded microring resonators, silicon photonics, photonic sensors, biochemical sensing

## Abstract

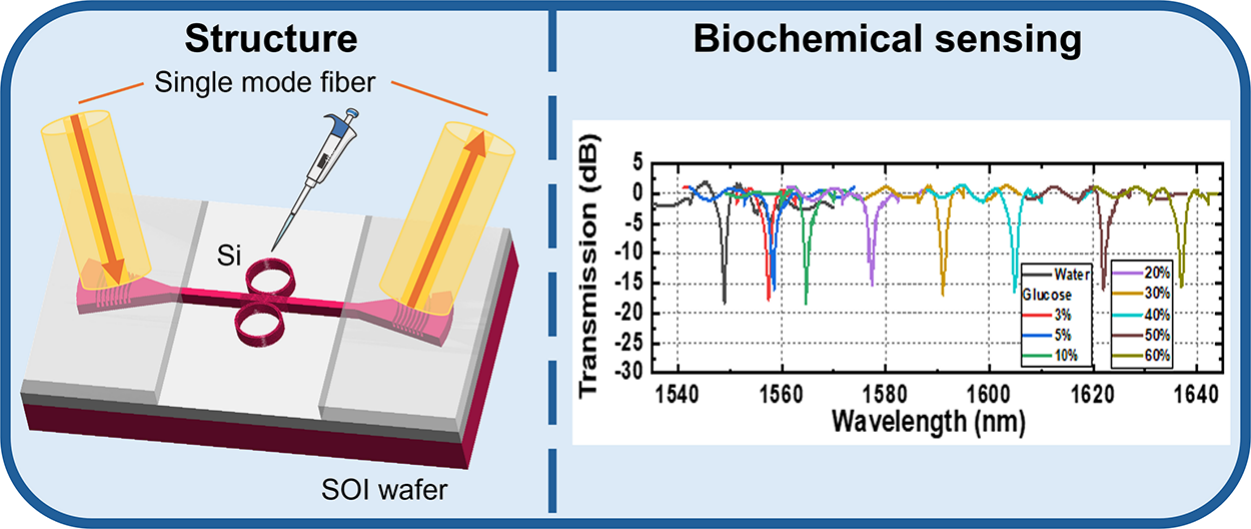

Photonic integrated circuit biochemical and biomedical sensors show promising applications in medical diagnosis, food security, healthcare, and environmental monitoring. Silicon-on-insulator subwavelength grating waveguides and cascaded microring resonator structures enhance photon-analyte interaction, offering superior sensing performance (higher sensitivity with lower limit of detection and larger free spectral range) compared to traditional strip and slot waveguide microring resonator structures. In this study, we design, simulate, and experimentally demonstrate a novel and compact biochemical sensor integrating subwavelength grating cascaded microring resonators and multibox subwavelength grating straight waveguides on a silicon-on-insulator platform. We achieve a record-high refractive index sensitivity of 810 nm/RIU with a limit of detection value of 2.04 × 10^–5^ RIU. The measured concentration sensitivity for sodium chloride solutions is 1430 pm/% with a limit of detection of 0.04%. The free spectral range is 35.8 nm, and the measured *Q* factor is 1.9 × 10^3^. By combining the advantages of cascaded microring resonators with those subwavelength gratings, our sensor offers unprecedented sensitivity for biochemical sensing applications, promising significant enhancements in healthcare diagnostics and environmental monitoring systems.

## Introduction and Background

Label-free optical methods are preferred for biochemical sensing due to their simplified experimental processes, which entail avoiding label-associated interference, enabling dense integration, and facilitating cost-effective fabrication, unlike traditional label-based detection strategies.^1^ Biosensors based on a silicon photonic platform are popular for continuous and quantitative label-free biosensing, facilitating simultaneous measurements on a single chip. Silicon photonics is a chip-scale technology focused on manipulating light at optical communication wavelengths in submicron silicon photonic wires.^2^ Its compatibility with mainstream complementary metal-oxide-semiconductor (CMOS) foundry processes facilitates the fabrication of complex, highly manufacturable, and compact chip-scale photonic systems for optical multiplexing,^3^ modulations,^4^ and biosensing applications.^5^ The significant refractive index (RI) contrast among the silicon waveguide, substrate, and cladding layers on the silicon-on-insulator (SOI) platform tightly confines and guides light, facilitating compact and economically scalable designs. This ensures large-scale and high-density photonic integrated circuit (PIC) design.

The evanescent field, as part of the electric field extending outside the silicon waveguide, is sensitive to changes in the RI outside the waveguide, such as analyte and binding molecules on the waveguide surface. Many label-free optical sensing devices on SOI wafer material platforms utilize resonant cavities such as microring sensors,^5^ microdisk sensors,^6^ grating sensors,^7^ and photonic crystal sensors.^8^ The label-free optical sensing mechanism involves attaching analytes or molecules to a waveguide surface. Their adsorption increases the local RI, leading to a change in the overall mode effective RI and triggering a resonant red shift of the cavity transmission spectrum.

For photonic biochemical sensors based on the microring resonators (MRR) structure using spectrum detection methods, two common interrogation techniques are employed: intensity interrogation and wavelength interrogation.^9^ The former has a narrow detection range and an unstable accuracy. Therefore, wavelength interrogation has become a popular detection method that meets the requirements of a large detection range and easy identification. Silicon photonic cavities have been used in a variety of clinically relevant applications, including the detection and identification of protein biomarkers,^10^ viruses,^11^ nucleic acids,^12^ and environmental toxins.^13^

Silicon photonic MRR structures have been extensively studied as biosensors due to their compact design and mature fabrication process. The commercially available silicon photonic biosensing platform (Genalyte) utilizes an MRR structure with TE-polarized light, provides a RI sensitivity of 54 nm/RIU, and has a typical bulk limit of detection (LOD) in the 10^–6^ RIU range.^5,14,15^ However, many clinical diagnostic assays require subsequent secondary binding events to obtain lower detection limits.^16,17^ Enhancing the sensitivity for clinically significant analytes poses challenges, such as ensuring robust surface chemistry (resistant to fouling and capable of accommodating high densities of captured molecules) and optimizing the intrinsic sensor properties. Numerous research groups are actively striving to enhance the sensitivity of MRRs.

To overcome the sensitivity limitations of current SOI MRR biosensors, many researchers have investigated waveguide structure improvements. Approaches employing strip waveguide TM mode MRR sensing^18^ (with a sensitivity of 270 nm/RIU) and slot waveguide MRR sensing^19^ (with a sensitivity of 563 nm/RIU) are encountering limitations in further enhancing sensitivity. Thus, designs adopting multislot subwavelength Bragg gratings and subwavelength gratings have been proposed to augment light-analyte interactions. Such designs have demonstrated significantly enhanced sensitivity levels, reaching 490^20^ and 730 nm/RIU,^21^ in contrast to conventional strip waveguide MRR sensors, which typically achieve 70 nm/RIU.^15^ Also, some proposed cascaded MRR structures show potentials in sensitivity improvement,^22^ reference sensing,^23^ and free spectral range (FSR) extension.^24^

This work introduces a novel, compact, and exceptionally sensitive label-free biochemical sensor. It utilizes a subwavelength grating cascaded microring resonator (SWG-CMRR) in conjunction with four rows of silicon multibox straight waveguide structures. Employing CMRRs in sensing offers numerous advantages including heightened sensitivity, tunable resonant wavelengths, and compact design. The gaps in transversal and propagation directions between silicon box segments significantly reduce the effective RI of the multibox waveguide optical mode and make the mode less confined. Also, this multibox structure provides more surface contact area for biomolecule attachment. Therefore, the sensitivity will be highly improved compared to that of traditional SWG-MRR sensors. The SWG-CMRR also exhibits notable superiority over the sidewall grating slot waveguide MRR (SG-SMRR)^25^ sensing structure design in terms of sensitivity, with an increase from 620 nm/RIU for the SG-SMRR to 810 nm/RIU for the SWG-CMRR. Also, it represents a sensitivity improvement compared to reported multibox MRRs.^26^ Additionally, the cascade setup can expand the FSR, broadening the range of wavelengths at which resonances can occur. However, it is crucial to recognize the challenges related to subwavelength grating fabrication, which include reactive-ion etching (RIE) lag effects and elevated waveguide loss due to the structure. In response to these challenges, our fabrication process is meticulously designed and optimized, ultimately achieving a high *Q* factor with a value of 1.9 × 10^3^ in the 5 μm radius SWG-CMRR structure. This is the first time that the ultracompact (5 μm radius) SWG-CMRR is fabricated, demonstrated, and reported. Taking into account the advantages and disadvantages previously discussed, our SWG-CMRR design combines SWG and CMRR structures to synergistically enhance sensitivity and extend the FSR. We achieved a record-high RI sensitivity of 810 nm/RIU by measuring various concentrations of sodium chloride as an analyte solution, with a corresponding LOD value of 2.04 × 10^–5^ RIU. Additionally, the measured concentration sensitivity is 1430 pm/%, with a LOD of 0.04%.

## Experimental Section

### Numerical Methodologies and Structural Design

**Figure 1:**
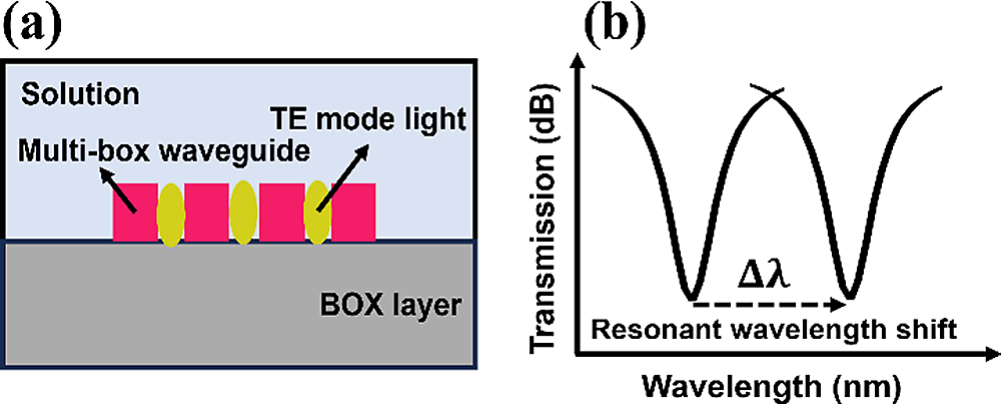
(a) Light–analyte interaction inside the multibox waveguide. (b) Schematic representation of resonance spectrum shifts due to changes in the effective refractive index of the guided mode.

The biochemical sensors based on the microring and multibox waveguide structure could detect analyte biochemicals with high sensitivity and selectivity with functionalized probe molecules. When the different concentrations of analyte solutions (glucose and sodium chloride) are attached to the slots inside the multibox waveguides, as shown in Figure 1a, they change the refractive index of the guided mode and cause a resonance wavelength shift shown in Figure 1b. Also, we can determine the concentrations of the analyte solutions via a calibration curve.

**Figure 2:**
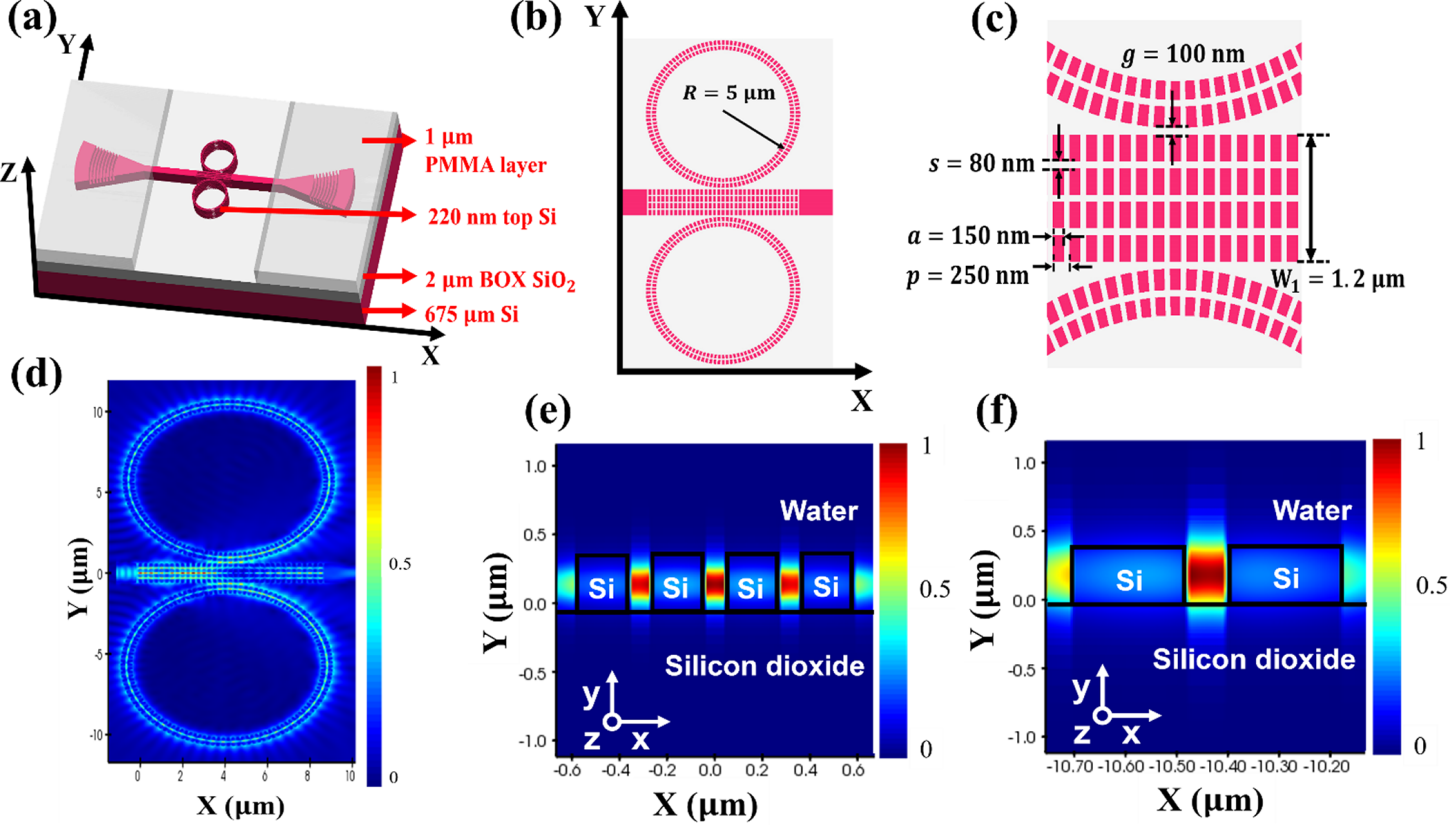
(a) Schematic structure of the SWG-CMRR sensor, (b) zoomed-in view of the SWG-CMRR, and (c) design parameters of the SWG-CMRR. Electric field distribution of the TE mode in (d) upper view of the SWG-CMRR, (e) side view of the multislot subwavelength Bragg gratings, and (f) side view of the subwavelength grating microring resonator (the color bar stands for electric field intensity).

The schematic of the proposed SWG-CMRR biochemical sensor is shown in Figure 2a. The reported numerical methods like Fourier eigenmode Expansion Method^27^ and bidirectional mode expansion and propagation method (BEP)^28^ can be used for periodic waveguide simulation. The full 3D vectorial finite-difference-time-domain (FDTD) approach among these simulation methods stands out for its rigor but can be time-consuming for large and intricate structures simulation. An alternative approach is to simulate a single unit cell using Bloch boundary conditions along the propagation direction (a method employed in SWG and Bragg waveguides simulation).^20^ This approach is effective for infinite long waveguide simulation without the entire structure modulation. The FDTD Solutions software from Lumerical Solutions, Inc. is employed for multibox waveguide and SWG-CMRR structure simulation.

The design of the SWG-CMRR sensor is based on a standard SOI wafer structure (provided by Soitec, Inc.). The wafer structure comprises a 220 nm top silicon layer, a 2 μm buried oxide (BOX) layer, and a 675 μm silicon substrate. A three-dimensional (3D) schematic of the SOI wafer and proposed SWG-CMRR sensor is illustrated in Figure 1. This SWG-CMRR biochemical sensor combines input and output grating couplers, CMRR, and multibox SWG waveguide structures.

The multibox SWG waveguides are designed with a period significantly smaller than the Bragg condition (Λ ≪ λ/2*n*_eff_). This ensures that the periodic waveguide structures support lossless Floquet–Bloch modes^29^ and theoretically support waveguide modes in SWG structures like conventional strip waveguides. However, due to the low optical confinement of the multibox structure, various additional losses need to be considered when introducing the multibox structure for sensitivity enhancement. The low optical confinement of the multibox waveguide introduces substrate leakage loss which is negligible when the thickness of the buried oxide (BOX) layer is higher than 2 μm and the mode effective index is higher than 1.65.^30^ Here, we incorporate our designed subwavelength grating structure into the loss analysis. The simulated mode effective index of our designed multibox subwavelength grating is 1.67, allowing us to disregard substrate leakage loss. Some additional sources of loss include mode mismatch, bending radiation, scattering, and material absorption. The additional scattering loss of the multibox waveguide is not negligible due to its multiple internal sidewalls. The two key factors that determine scattering process properties are the correction length of disorder (*L*_C_, the distance between one to another correlated defect) and root-mean-square roughness (σ). The value of *L*_C_ is 50 nm and σ is less than 2 nm for typical SOI fabrication technology.^25^ High sensitivity is achieved through the utilization of multislot SWG and SWG-MRR structures, enhancing the interaction between light and analytes. Efficient light coupling between the sensing device and cleaved single-mode fibers (SMF) is achieved using focused grating couplers.

The detailed design parameters of the SWG-CMRR sensor are shown in Figure 2b,c. The radius of the SWG-CMRR is defined as the distance from the center of the ring to the outer radius of the inner SWG-CMRR, set at a value of *R* = 5 μm. The width of the straight multibox waveguide is defined as *W*_1_ = 1.2 μm, with a segment length of *a* = 150 nm and a grating period of *p* = 250 nm. The fill factor (FF) is 0.6, calculated as the ratio of the multibox segment length to the grating period. The multibox straight waveguide cavity length is 8.86 μm. The slot width between each row of multibox gratings is set to *s* = 80 nm, ensuring both increasing the interaction area for light-analyte sensing and allowing the smallest definition pattern of the electron beam lithography (EBL). The SWG in the MRR has the same grating segment width and grating period as the straight multibox waveguide. The gap between the straight multibox waveguide and the SWG-CMRR is set at *g* = 100 nm. The multibox grating period *P*, FF, and slot width *s* have been meticulously designed for optimal performance, aiming to maximize the light intensity confined within the subwavelength grating slots. This design ensures an enhanced interaction between light and analyte, specifically tailored for improved sensing applications. The electric field distributions of the TE-guided mode with water cladding layer in the SWG-CMRR sensor, the multislot SWG, and the SWG-CMRR are shown in Figure 2d–f, respectively. These figures depict simulation results based on our design parameters, demonstrating significant light-intensity confinement within the slots. As shown in Figure 2e, the optical confinement factors within the three slots of the multibox straight waveguide are 31.6, 35.3, and 27.7% from left to right, respectively. Additionally, as shown in Figure 2f, the optical confinement factor within the slot of the subwavelength grating microring resonator waveguide is 55.2%.

One potential challenge with manufacturing the SWG-CMRR involves the fabrication difficulties associated with etching the slots required to form the multibox silicon subwavelength gratings, particularly without encountering significant RIE lag effects. Fortunately, our fabrication techniques can realize slot widths as narrow as 80 nm without such issues.

### Measurement Setup

**Figure 3:**
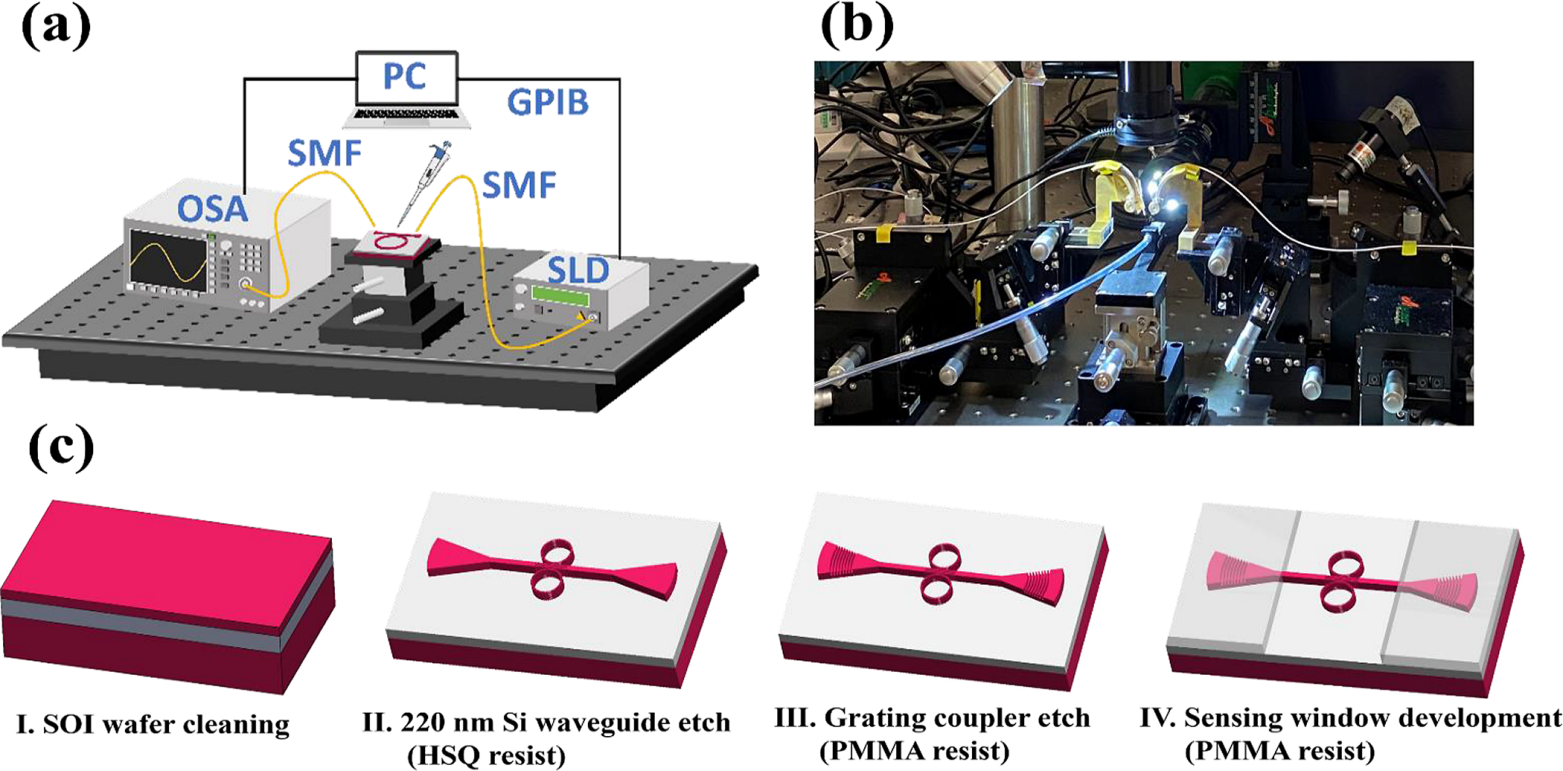
(a) Schematic diagram of the experimental setup, (b) photograph of the measurement stage, and (c) fabrication steps of the device.

The fabricated SWG-CMRR devices were measured and characterized using a silicon photonic test setup (vertical coupling based) and LABVIEW software developed by our research group for biochemical sensing applications. The experimental setup for the characterization of the SWG-CMRR biochemical sensors is illustrated in Figure 3a,b.

In the experiment setup, a super luminescent diode (SLD, THORLABS S5FC1005P - PM Benchtop SLD Source) with a central wavelength of 1550 nm, a 3 dB bandwidth of 50 nm, and a maximum output power of 22 mW was used as the broadband light source for transmission spectrum measurement of the biochemical sensors. The optical spectrum analyzer (OSA) with a resolution bandwidth (RBW) of 0.06 nm is used to measure the transmission spectrum response of sensors. The six-axis coupling stages are used for coupling efficiency optimization between the SMFs and grating couplers (GCs). The input and output light beams in TE mode were coupled to the SWG-CMRR sensors via 10 μm core cleaved SMFs with 10° tilted angles from the vertical direction for both input and output angles through the GCs.

To showcase the sensitivity properties and parameters, we apply various concentrations of analyte solutions onto the SWG-CMRR sensor, measuring the resonant peak shift in the transmission spectrum. Subsequently, different sensitivity parameters are calculated based on the acquired data. A range of solutions, including various concentrations of glucose and sodium chloride, are employed in laboratory measurements. These solutions are dropped onto the sensing MRR section, as illustrated in Figure 3a. The RI values for different concentrations of glucose and sodium chloride solutions at a wavelength of 1550 nm are detailed in a previously reported paper.^19^ An automated measurement system was developed utilizing the general-purpose interface bus (GPIB) connection to interface with measurement devices controlled by LABVIEW software. This setup enables rapid data acquisition, which is particularly crucial for solvents that evaporate quickly.

## Results and Discussion

### Fabrication of the SWG-CMRR

The fabrication process for the SWG-CMRR sensor is illustrated in Figure 3c. It comprises four main steps, akin to the detailed descriptions provided in reference.^19^ However, there are specific differences in certain steps, which are detailed here. First, the cleaved 11 × 12 mm^2^ SOI wafer underwent cleaning by immersion in acetone, isopropanol (IPA), and reverse osmosis (RO) water with an ultrasonic bath. Second, one layer of electron beam lithography (EBL) resist was used to define silicon multibox and subwavelength grating waveguide patterns (1:3 HSQ: MIBK, baked at 90 °C, 25% tetramethylammonium hydroxide PMMA development). After that, the top silicon multibox and subwavelength grating microring patterns were dry etched using an inductively coupled plasma (ICP) SPTS Rapier DSiE with flow rates of C4F8/SF6 (90:30 sccm). Third, another layer of electron beam lithography (EBL) resist was used to define the focused GC patterns (AR-P 642 200k Anisole 12% polymethylmethacrylate PMMA, baked at 180 °C, 2.5:1 IPA: MIBK development). Then the focused GC patterns were dry etched using an inductively coupled plasma (ICP) SPTS Rapier DSiE with flow rates of C_4_F_8_/SF_6_ (90:30 sccm). Fourth, it is noteworthy that the step differs from the reported reference.^19^ Instead of a 1 μm silicon dioxide layer, one PMMA resist cladding layer is utilized as an alternative for enhancing the GC coupling efficiency. This modification aims for a streamlined and optimized fabrication process, which is particularly beneficial for rapid testing and short fabrication cycles. The resist layer is selectively exposed, developed, and removed to create an open window above the SWG-CMRR sensors, facilitating their sensing functionality. Here we note that the EBL resist thicknesses, doses, and beam step size (BSS) were carefully optimized based on dose tests and fabrication tests to produce a high-resolution subwavelength grating, smooth sidewalls, and well-defined gaps between the silicon multibox elements. As a result, the deviation between the designed and fabricated dimensions is minimal.

**Figure 4:**
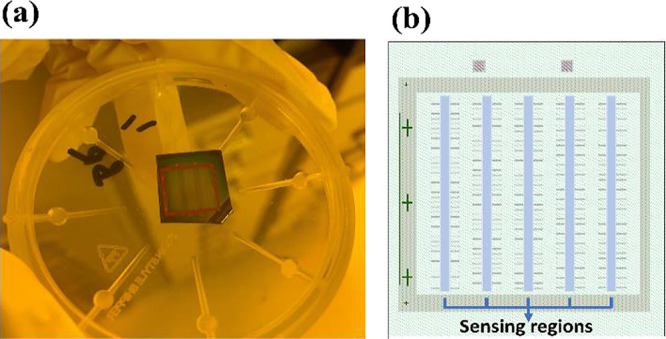
(a) Photograph of the miniature device. (b) Sensing open window channels for experimentation.

The SOI sample wafer, featuring five columns of biochemical sensors with various design structures, is fabricated in a single fabrication run, enhancing the efficiency in both fabrication and testing. Figure 4a depicts a photograph of the test wafer captured in the clean room laboratory. The sensing channel regions of the sensor wafer are highlighted in Figure 4b.

**Figure 5:**
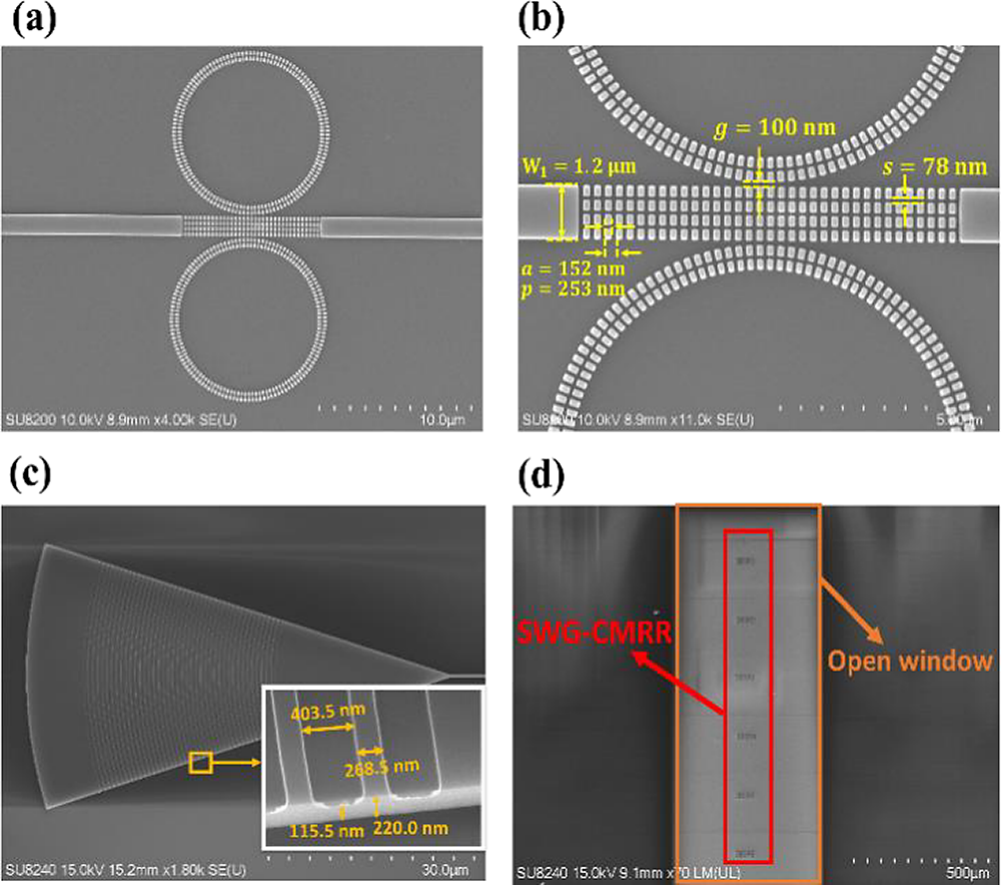
(a) SEM images of the SWG-CMRR, (b) zoomed-in SWGs with fabrication parameters, (c) GC, and the inset shows the grating period of 671 nm and dry etched grating height of around 105 nm, and (d) open window for a series of SWG-CMRRs.

While the wafer size is initially 11 × 12 mm^2^ for research project testing, it can be tailored for mass-scale photonic integrated circuits, accommodating 4- or 6-in. wafer structures. The top-view scanning electron microscopy (SEM) image of the fabricated SWG-CMRR device is depicted in Figure 5a. Figure 5b presents a zoomed-in SEM image of the SWG multibox structure with measured fabrication parameters, while Figure 5c showcases the fabricated GC. Additionally, Figure 5d displays the open window on the SWG-CMRR for high-sensitivity analyte sensing.

For SWG-CMRR fabrication, the dimensions of the multibox straight waveguide (*W*_1_) are set at 1.2 μm, with a gap (*g*) of 100 nm between the straight multibox waveguide and the SWG-MRR. The multibox segment length (*a*) is 152 nm, the multibox grating period (*p*) is 253 nm, and the slot width (*s*) between each multibox grating row is 78 nm. These dimensions were designed as *W*_1_ = 1.2 μm, *g* = 100 nm, *a* = 150 nm, *p* = 250 nm, and *s* = 80 nm. The period and duty cycle of the fabricated grating coupler are measured at 672 and 40%, respectively, with an etch depth of 105 nm. These fabricated parameters closely align with the designed period and duty cycle of 671 nm and 39.9%, respectively. The measured central wavelength and coupling efficiency of the fabricated grating couplers are 1555 and 41%, respectively, compared to 1555 nm and 44% in simulation. Slight parameter differences observed between the design and fabrication may stem from fabrication errors, such as resist development variations, proximity effects in EBL, and sidewall etching during the ICP-RIE process.

### Real-Time Sensing Performance in Analyte Solutions

**Figure 6:**
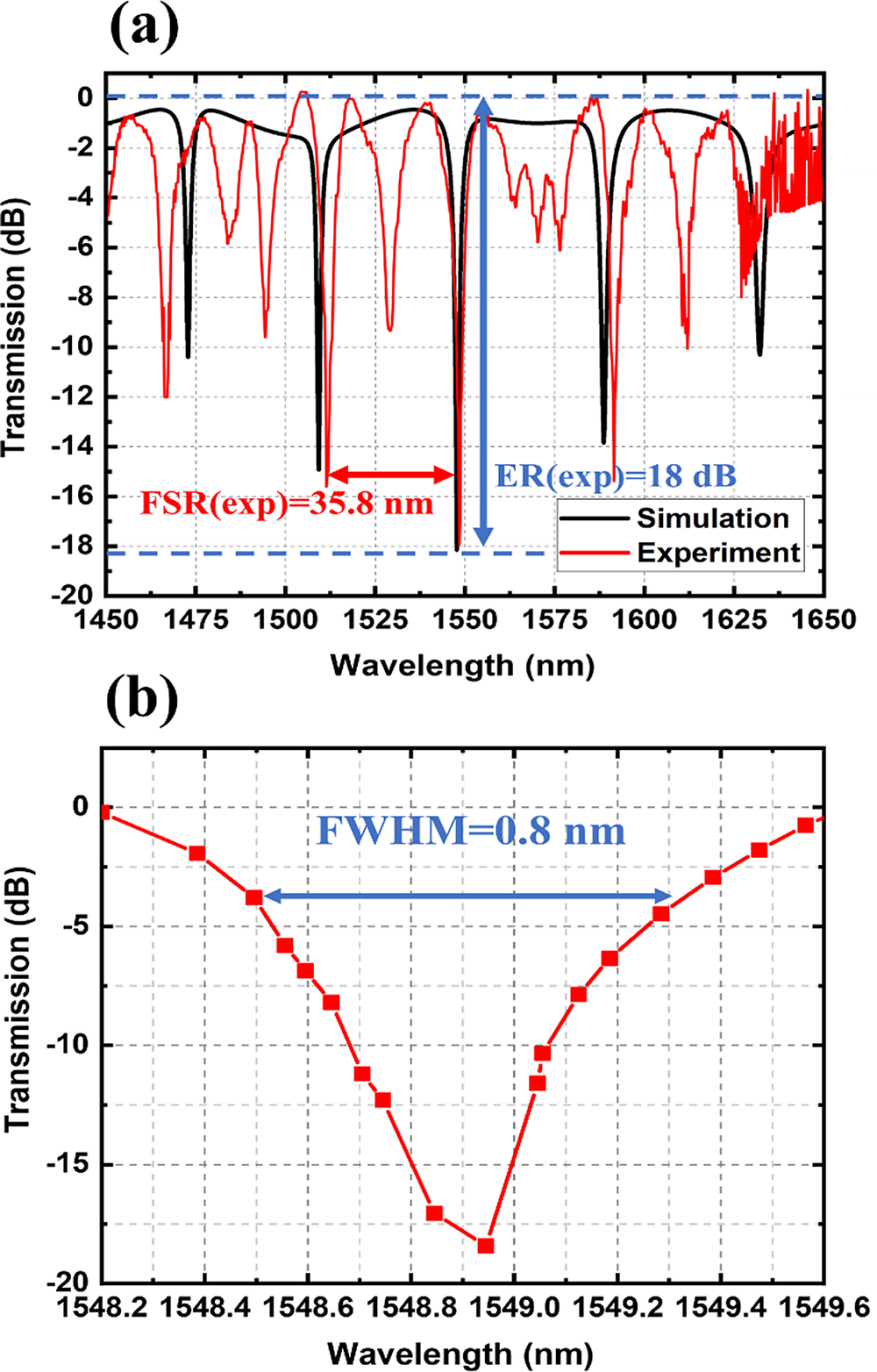
(a) Simulated (black) and measured (red) SWG-CMRR transmission spectra in water. (b) Zoomed-in-monitored resonant peak of the SWG-CMRR transmission spectrum in water.

The simulated and experimental transmission spectra of the fabricated SWG-CMRR biosensor are shown in Figure 6a, along with the zoomed-in resonant peak shown in Figure 6b. The measured spectra are conducted with a water envelope environment.

A set of glucose and sodium chloride solutions with different concentrations ranging from 3 to 60% for glucose and 3 to 25% for sodium chloride were selected as the refractive index standards for bulk sensitivity measurements. Since these analyte solutions are soluble in water and volatile at room temperature, no residues are left on the silicon chip after rinsing, cleaning, and drying with a nitrogen gun. During optical measurements, the stage was thermally adjusted and maintained at room temperature to minimize the effects of external thermal noise and drift. Each SWG-CMRR sensor took multiple measurements of each concentration to ensure signal stability and accuracy.

The resonant peak monitored for biochemical sensing starts at a 1548.9 nm wavelength (the simulated resonant peak is at 1547.7 nm). The monitored resonant peak can be tuned from 1548.9 to 1650 nm in response to analyte solutions with different RIs. The measured resonant peak is observed with a 1 nm redshift compared to the simulation result. This difference may be attributed to variations in the fabrication and silicon etch thickness relative to the specified design parameters. The measured FSR of the SWG-CMRR is 35.8 nm. This measured value is less than the simulated FSR of 39.8 nm. The 4 nm shrink variation observed between the simulated and measured FSR values can be ascribed to factors arising during fabrication. These factors include (1) an elevation in the group index resulting from the etched ridge height being only 210 nm as opposed to the simulated height of 220 nm. The presence of a 10 nm unetched silicon layer on either side of the ridge would lead to a 1.2 nm reduction in the FSR. (2) Small deviations in the FF of the subwavelength grating during resist development, for example, an increase of only 1% in FF would result in a 1.1 nm reduction in FSR.

Also, we noticed that there are some low extinction ratio (ER) resonant peaks between the main MRR resonant peaks in the measured SWG-CMRR transmission spectrum. This could be attributed to reflections occurring inside the straight waveguide cavity. This hypothesis is supported by the calculation of the FSR of the straight waveguide cavity (with a total straight waveguide cavity length of 28.8 μm and a group index of 2.7). The FSR of a straight waveguide cavity is 15.4 nm, which is close to the transmission result. Furthermore, the measured insertion loss of the strip to multibox waveguide transition is 0.8 dB. This loss aligns with the ER between the main MRR peaks and the parasitic peaks in the transmission spectrum. The resonant peak is at a 1548.9 nm wavelength with a full-width half-maximum (fwhm) value of 0.8 nm. The measured ER value for the monitored resonant peak is 18 dB, closely matching the simulated ER of 18.1 dB.

**Figure 7:**
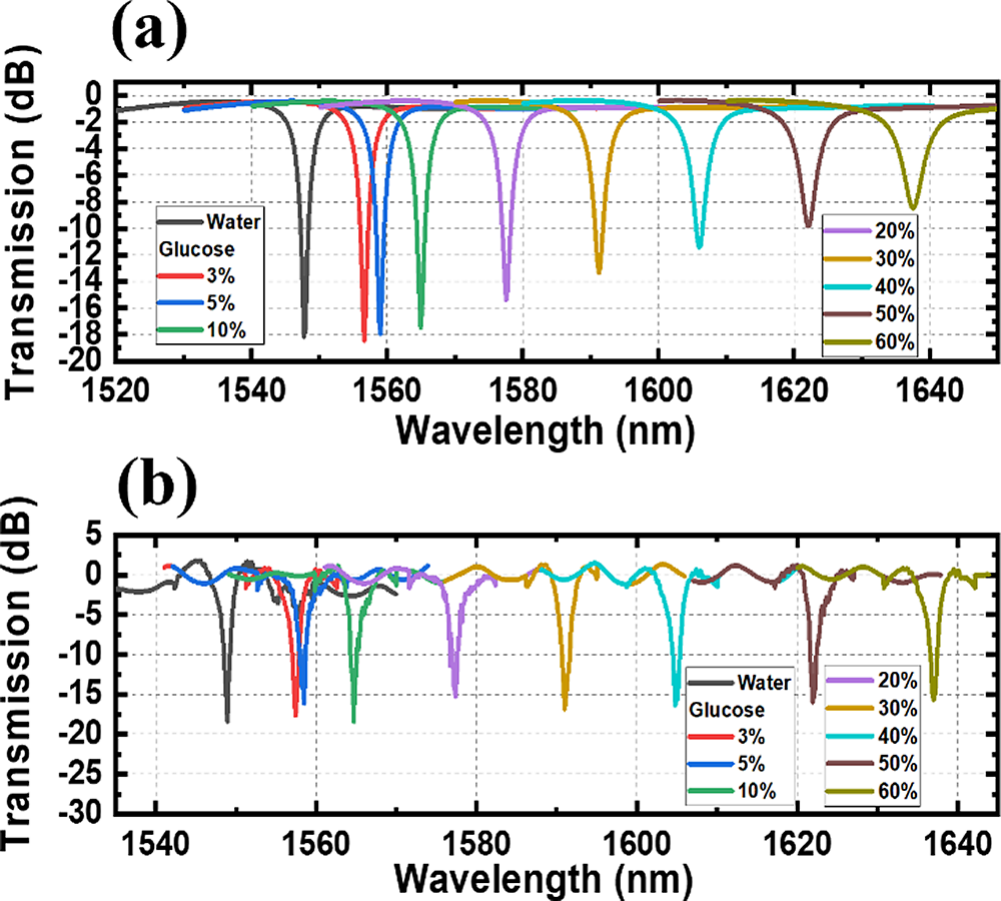
Simulated (a) and measured (b) transmission spectra of different concentrations of glucose solutions.

**Figure 8:**
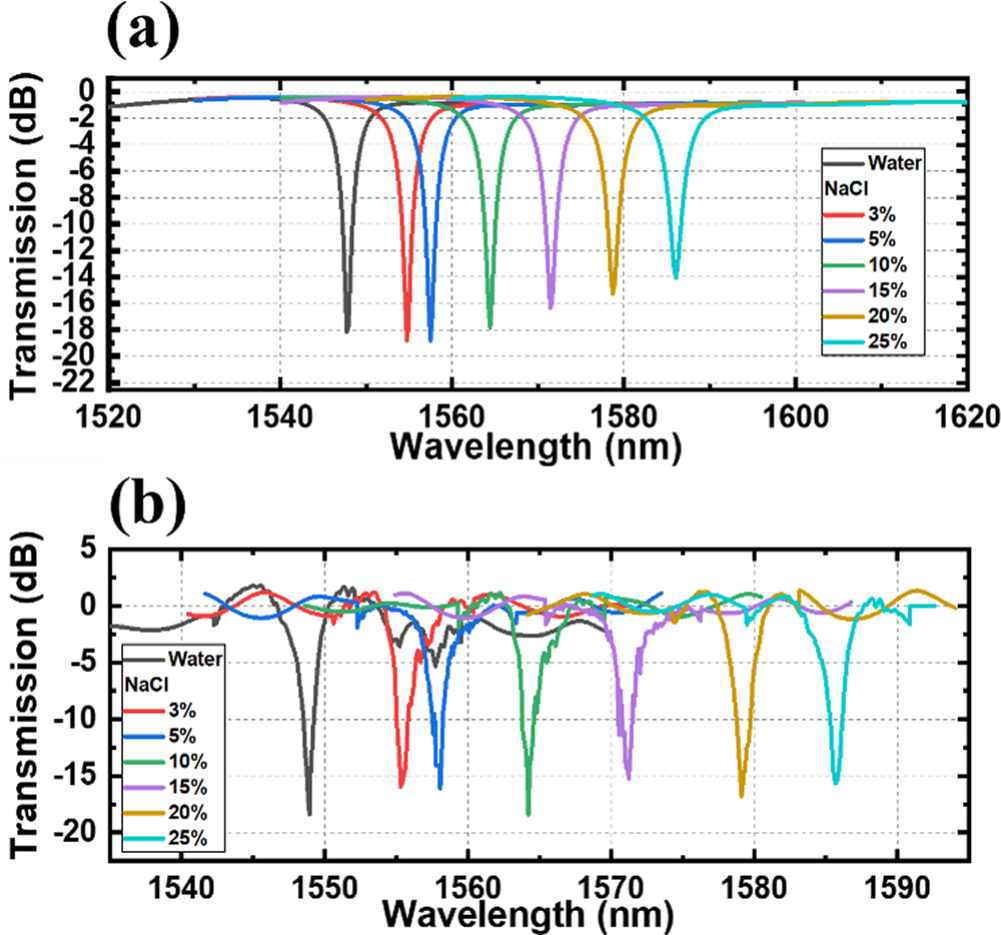
Simulated (a) and measured (b) transmission spectra of different concentrations of sodium chloride solutions.

**Figure 9:**
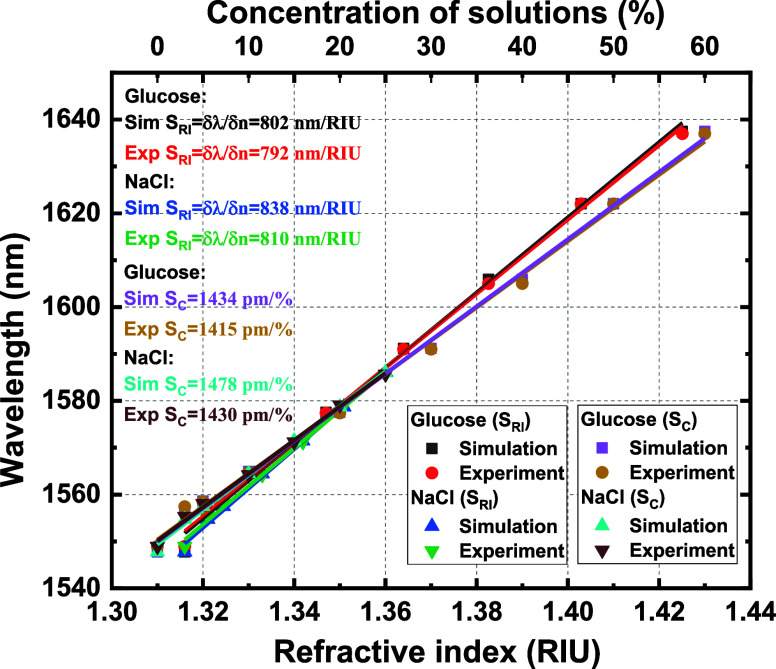
Simulated and experimental RI sensitivities and concentration sensitivities of SWG-CMRR for glucose and NaCl solutions.

The monitored resonant peak red shift with different concentrations of glucose solutions in the envelope environment in simulation and measurement is shown in Figure 7a, b, respectively. For different concentrations of glucose solutions, the concentrations of the solution change from 3 to 60% (RI changes from 1.32 to 1.43). The monitored resonant peak started at 1548.9 nm and red-shifted to 1637 nm in measurement (starts from 1547.7 to 1637.5 nm in simulation). The monitored resonant peak red shift with different concentrations of sodium chloride solutions in the envelope environment in simulation and measurement is shown in Figure 8a, b, respectively. For different concentrations of sodium chloride solutions, the concentrations of solution change from 3 to 25% (RI changes from 1.32 to 1.36). The monitored resonant peak initially measured at 1548.9 nm, shifting to 1585.7 nm during the experiment (while it began at 1547.7 nm and extended to 1586.1 nm in simulation). The monitored resonant peak red shift with different concentrations of glucose and sodium chloride solutions in the envelope environment in simulation and measurement is shown in Figure 9. Various sensitivity parameters (refractive index sensitivity with corresponding LOD, concentration sensitivity with the related LOD) can be computed and analyzed. The LOD is defined as the resonance wavelength resolution 3σ divided by the sensitivity (concentration sensitivity *S*_C_ or refractive index *S*_RI_), where σ is the standard deviation of the resulting spectral variation. Sensitivity calculation entails a linear regression of wavelength peak data, while the LOD is determined using the formula detailed in the referenced report.^19^

The simulated and measured RI sensitivities (*S*_RI_) of the SWG-CMRR sensor of different glucose and sodium chloride concentrations are shown in Figure 9. The simulated and measured RI sensitivity of different concentrations of glucose solutions are 792 and 802 nm/RIU, respectively. The simulated and measured RI sensitivity of different concentrations of sodium chloride solutions are 838 and 810 nm/RIU, respectively. Thus, the highest measured RI sensitivity we could derive from the experiment is 810 nm/RIU. Additionally, this RI sensitivity value is accompanied by a record-low LOD value of 2.04 × 10^–5^ RIU.

Here, it is important to note that theoretically, the measured bulk sensitivity for glucose and sodium chloride solutions should be equal. However, observed differences in measured sensitivities using the SWG-CMRR may arise from various factors. Variations in experimental conditions, such as temperature fluctuations and mechanical vibrations or uncertainties in the measurement process, could contribute to these differences. Another potential factor could be the limited RBW of the OSA (0.06 nm).

The simulated and measured concentration sensitivities (*S*_C_) of the SWG-CMRR sensor of different glucose and sodium chloride concentrations are also shown in Figure 9. The simulated and measured concentration sensitivity of different concentrations of glucose solutions are 1434 and 1415 pm/%, respectively. The simulated and measured concentration sensitivity of different concentrations of sodium chloride solutions are 1478 and 1430 pm/%, respectively. Thus, the highest measured concentration sensitivity we could derive from the experiment is 1430 pm/%. Additionally, this concentration sensitivity value is accompanied by an LOD value of 0.04%.

## Conclusions

In conclusion, a highly sensitive and compact SWG-CMRR with a radius of 5 μm was successfully designed, fabricated, and experimentally demonstrated. Both simulation and experimental results reveal that the optical power is predominantly concentrated in the gaps between silicon multibox segments, significantly enhancing the overlap between the evanescent field and the analyte. The recorded RI sensitivity, utilizing the SOI MRR structure, reaches 810 nm/RIU, with a corresponding LOD value of 2.04 × 10^–5^ RIU. Notably, the concentration sensitivity and minimum concentration detection limit stand at 1430 pm/% and 0.04%, respectively. The measured FSR in this SWG-CMRR structure is 35.8 nm. The fabrication work, involving dose optimization, ridge waveguide edge smoothing, and resist development optimization in EBL pattern preparation, results in an improved Q factor of the subwavelength grating structure, with a value of 1.9 × 10^3^. These features collectively establish the SWG-CMRR as a highly promising candidate for diverse applications including sensing, environment monitoring, and biomedical diagnostics. Its capability for precise and efficient detection of changes in the refractive index and concentration renders it particularly valuable in these fields.
